# Selective Strategy Differential Evolution for Stochastic Internal Task Scheduling Problem in Cross-Docking Terminals

**DOI:** 10.1155/2022/1398448

**Published:** 2022-11-04

**Authors:** Dollaya Buakum, Warisa Wisittipanich

**Affiliations:** ^1^Department of Industrial Engineering and Manufacturing, Faculty of Engineering, Prince of Songkla University, 15 Karnjanavanich Road, Hat Yai, Songkhla 90110, Thailand; ^2^Advanced Manufacturing and Management Technology Research Center (AM2Tech), Department of Industrial Engineering, Faculty of Engineering, Chiang Mai University, 239 Huay Keaw Road, Suthep, Muang, Chiang Mai 50200, Thailand; ^3^Supply Chain and Engineering Management Research Unit, Chiang Mai University, Chiang Mai 50200, Thailand

## Abstract

This study proposed an algorithm called selective strategy differential evolution (SSDE) to handle the complexity of the stochastic internal task scheduling problem in cross-docking terminals. The aims of this study are to assign workers and transfer equipment to internal operations and sequence those operations under randomness and uncertainty with the purpose to minimise total tardiness. The main feature of SSDE is its ability to adapt itself in order to execute the best search strategy. The proposed algorithm was tested on 16 instances using generated data based on real-case scenarios of a pharmaceutical distribution centre. The results showed the significant performance of SSDE to other existing algorithms in terms of solution quality and computational time. The key success factors of SSDE are the use of various search strategies in a single run and the application of suitable termination conditions.

## 1. Introduction

Warehouse management problem has driven researchers to seek the least-cost operation which optimize ordering and holding costs and maximize space utilization by leveraging existing technologies. A cross-docking strategy is one of the logistics practice of unloading goods from incoming trucks and loading them directly into outbound trucks with little to or storage in between. Since cross-docking does not involve storing goods in the warehouse, costs associated with handling and storage are reduced and deliveries are faster. In addition, cross-docking enables greater throughput without the need for opening up a new warehouse. Undeniably, insufficient operational management in cross-docking may obstruct its successful implementation. Recently, studies concerning operation problems in cross-docking have been addressing the real-world problem or specific conditions. Rijal et al. [1] studied truck scheduling and dock door assignment at unit-load cross-dock terminals where dock doors can operate in a mixed service mode. Zheng et al. [2] addressed the cold-chain cross-docking truck scheduling problem. Shahmardan and Sajadieh [3] investigated a truck scheduling problem at a cross-docking centre where inbound trucks were also used as outbound and they can be partially unloaded. Correa Issi, Linfati, and Escobar [4] proposed a mathematical model for truck scheduling in cross-docking in a mixed service mode dock area of a multinational food company in Chile. Chargui et al. [5] proposed a mathematical model to optimize the scheduling, storage, assignment, and sequencing of trucks at receiving and shipping docks for a problem inspired from a multiple door cross-dock facility of an industrial partner with multiple temporary storage zones. However, there exist some limitations to the aforementioned references. One of the common suggestions from those articles is to incorporate stochastic considerations into the problem to provide more practical and novel directions for future studies. In addition, adding a resource-constrained in the model was suggested by Shahmardan and Sajadieh (2020) as a direction for future research.

Task environments in a real-world practice are subject to several uncertainties and randomness. Therefore, a practical model for scheduling problems should be established to address uncertainty issues. Picking orders is typically discussed under the assumptions that processing times and due dates are known in advance and machines are continuously available. However, in practice, some of these assumptions in man-to-goods picking systems are unrealistic. In fact, workers may have different speeds owing to fatigue or other reasons. In addition, workers moving between picking locations can disrupt and lock each other. The phenomenon of pickers locking results in the time loss arising from waiting for the ability to continue the picking process [6].

**Figure 1:**
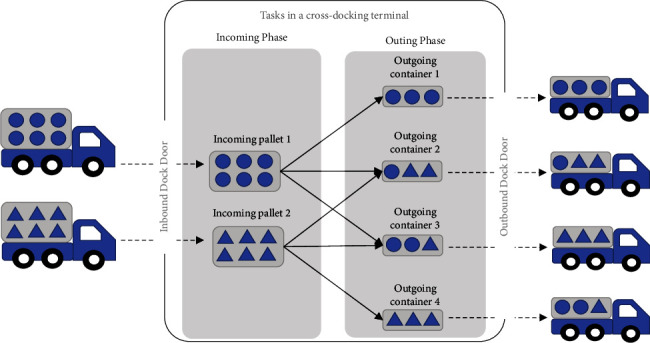
Tasks for a single unloading activity in a cross-docking terminal.

The stochastic internal task scheduling problem is a more practical model to deal with uncertainties and randomness in a real-world environment. This consideration could fill the current research gap in the cross-docking platform. To date, there are few studies on the stochastic internal task scheduling problem in a cross-dock terminal. Buakum and Wisittipanich [7] proposed a mathematical model for stochastic internal task scheduling to minimise the total tardiness of customer orders. The goal of their study was to simultaneously assign internal cross-docking workers and transportation equipment to obtain the optimal internal task schedule in a single unloading activity.  Figure 1 represents a single unloading activity in a cross-docking terminal. In the cross-docking terminal, the cross-docking operations can be divided into two phases: incoming and outgoing phases. In the incoming phase, the containers reach the cross-dock with different products. Each incoming container is processed by a breakdown operation such as scanning or sorting processes. In the outgoing phase, customers' orders are built up. Each customer order is processed by a build-up order operation, and some orders may require value-adding services, such as repacking or labelling.

In 2020, Buakum and Wisittipanich formulated a mathematical model of stochastic internal task scheduling problems in a cross-docking terminal using chance-constrained programming. In their study, an exact method using an optimisation solver was employed to obtain the optimal solution. The preliminary results showed that the problem was NP-hardness (nondeterministic polynomial-time hardness). The model consumed very high amount of computational time when the problem size slightly increased, and it failed to solve a large-scale case of the real-world problem. As a result, the exact method was not robust for dealing with uncertainty of processing time and due date.

Thus, this study focuses on metaheuristic applications to obtain a near-optimal solution within an acceptable computing time. This application aims to apply the stochastic model proposed by Buakum and Wisittipanich [7] in order to schedule large-scale internal tasks for timely medicine delivery to prevent negative effects on patients. Taking inspiration from the aforementioned problem statements, differential evolution (DE), a type of stochastic search and optimisation method, is used in this study. Starting from a population of randomly initialised solutions, the original DE algorithm employs simple mutation and crossover operators to generate new candidate solutions. Therefore, the performance of the DE algorithm is sensitive to the mutation and crossover schemes. Recently, various DE strategies with new mutation and crossover schemes have been introduced to enhance DE searching ability. However, a large amount of time is required to determine the appropriate DE evaluation strategies for specific problems under consideration.

To overcome this dilemma, this study proposes a novel self-adaptive and strategy selection-based DE technique called selective strategy differential evolution (SSDE). The main contributions of this study are listed as follows:

- The robust methods of the SSDE algorithm is proposed for dealing with uncertainty of processing time and due date; thus, the SSDE can properly handle large-scale internal task scheduling problems in a cross-docking terminal.
- An intelligible modification procedure that transforms classical DE to the SSDE is presented in this work. In addition, the application of suitable termination conditions is implemented in SSDE.
- The development of encoding and decoding procedures is proposed for finding the optimal order-picking operation in a single unloading manner under stochastic environments.

The remainder of this paper is organised as follows: Section 2 reviews related studies on the internal task scheduling problem in a cross-docking terminal. This section also reviews the modification of the classical DE algorithm and DE applications in cross-docking.  Section 3 presents the mathematical model of the problem.  Section 4 describes three classical metaheuristics used for experimental comparison in this study.  Section 5 introduces the SSDE algorithm, and the solution representation of the encoding and decoding schemes is described in Section 6. A computational experiment using scenarios from a real-case study is reported in Section 7. Finally, the conclusion is provided in Section 8.

## 2. Related Studies

The internal task scheduling problem in cross-dock terminals was first studied by Li et al. [8]. The problem was considered to be a parallel machine scheduling problem. The mathematical model was formulated with the objective of just-in-time shipments, and the resource constraint in the model was limited to the number of workers. Then, two heuristic algorithms such as squeaky wheel optimisation embedded in a genetic algorithm (SWOGA) and linear programming within a genetic algorithm (LPGA) were implemented to solve the problem and compared with solutions obtained from a CPLEX solver. The comparison results showed that the CPLEX solver has limited memory for solving the problem, whereas metaheuristic methods can provide near-optimal solutions for all test problems. Later, Alvarez-Perez et al. [11] proposed another metaheuristic, the reactive greedy randomised adaptive search procedure and tabu search (RGTS), to solve the same problem as that of Li et al. (2004). The results showed that the RGTS obtains better objective values and less computational time than the SWOGA and LPGA in some instances.

Workers and transferring tools are both important resources for internal tasks in cross-docking terminals in real-world problems. Recently, the developed mathematical model of internal task scheduling in cross-dock terminals was proposed to simultaneously assign workers and transfer equipment for operations to minimise the makespan [12]. The model was formulated in a deterministic environment; however, tasks in the real-world were operated under a stochastic environment. Therefore, Buakum and Wisittipanich [7] transformed the deterministic model into a stochastic model by using chance-constrained programming to minimise the total tardiness, in which the processing times and due dates of operations were considered as stochastic parameters. An exact method using the LINGO optimisation solver was used to find the optimal solution for the generated data. The results showed that LINGO required high computational time and was terminated with memory insufficiency in a large-scale case of the real-world problem. Several research studies have considered the internal task scheduling problem as a parallel machine scheduling (Li et al. [8] [7, 11, 12]). Afshar-Bakeshloo et al. [9] considered the internal task scheduling problem as single machine scheduling while Hamdi and Tekaya [10] considered the internal task scheduling as flow shop scheduling in deterministic environment.

A classical DE was first suggested by Storn and Price [13], which proved its high performance in solving scheduling problems [14–17]. In addition, better DE performance was observed when compared to other evolutionary algorithms (EAs), such as PSO on a suite of 34 widely used benchmark problems [18]. Nevertheless, low convergence speed is a problem of a classical DE, which requires high computational efforts. To overcome this restriction, several variations of the DE algorithm have been suggested to improve the performance of DE by modifying the evolution process during the search or integrating some additional processes into the DE to increase the convergence rate, such as local search [19], external archive [20], or antiaging mechanisms [21]. Pant et al. [22] presented an extensive survey of existence of DE. A number of research articles have been shown through variants of differential evolution like initialization techniques, modifications in mutation schemes, modified crossover schemes, modifications done in selection schemes, changes in parameters, and hybrid variants of different evolution.

Consequently, in terms of adaptive DE variants, different adaptation mechanisms have been introduced to dynamically update the DE algorithm to reproduce new generation of vectors without any prior knowledge of the relationship between the algorithm and the characteristics of optimisation problems. The adaptation mechanisms in the DE algorithm can be categorized into three classes as follows.

### 2.1. Parameter Adaptation

The performance of DE highly depends on the control parameters in the mutation operator, scaling factor *F*, the crossover operator, and crossover rate *CR.* Therefore, some studies have focused on the modification of control parameters in mutation and crossover operations [20, 23–28]. For example, Huynh et al. [28] proposed *Q*-learning model; the parameter controller adaptively adjusts the algorithm parameters at runtime using information from previous iterations.

### 2.2. Strategy Adaptation

In 2005, Price et al. [29] proposed other variants of DE derived from the different strategies of mutation and crossover schemes. However, a significant amount of time was required to find the best strategies for each specific problem. In order to automatically select the most suitable strategy while solving a problem without any prior knowledge, some approaches were introduced to apply multistrategies in a single run [30–33]. In the strategy adaptation class, parameter adaptive ability and/or other additional abilities could be implemented along with strategies adaptive ability [34–47]. For example, Do et al. [46] proposed a modified DE by adjusting scale factor F and crossover rate *c* as well as the mutation and selection phases of the original DE are also replaced by the best individual-based mutation and elitist selection techniques. In addition to adaptive mutation or crossover phases, the adaptive initialization phase was considered in this class. For example, Tang and Lee [47] proposed a straightforward and effective scheme for adaptive initialization and coupled with a linear reduction to the DE population size [26], called L-SHADE, for solving constrained optimisation problems.

### 2.3. Algorithm Adaptation

Since several DE variants have been proposed in recent years, some studies proposed approaches to employ multiple state-of-the-art DE variants for solving particular problems [48, 49].

According to the aforementioned references, Buakum and Wisittipanich [33] proposed the modified DE algorithm in the strategy adaptation class which contained both different mutation and crossover schemes in the strategy pool called self-learning differential evolution (SLDE) algorithm. The adaptive ability of SLDE was made by the ranking and updating probability of each constituent strategy in each generation. Thus, both the mutation and crossover operators were adaptable, and the strategy assignment of the proposed algorithm was based on one active strategy. However, SLDE was designed in deterministic aspect only.

To present a more practical problem and provide novel directions for addressing this problem, this study consider the stochastic internal task scheduling problem and proposed the modified DE algorithm which extends the SLDE in the stochastic aspect. Then, five metaheuristic algorithms, GA, PSO, DE, RAM-EPSDE, and SSDE are applied in the comparison experiments.

## 3. Mathematical Model of the Problem

The problem statement and a mathematical model were taken from Buakum and Wisittipanich [13]. The problem was formulated to minimise the total tardiness of the order-picking operation according to prescriptions in a single unloading activity. In a stochastic environment, processing times and due dates were set as random parameters, and their distributions were assumed to be known in advance. Based on the limited number of workers and transfer equipment, the goal of the formulation was to obtain an optimal schedule to simultaneously assign workers and transfer equipment for handling medicine pallets and prescriptions. The due date of each prescription was set based on the delivery date and patient location. The basic assumptions of the mathematical model were shown as follows:

- All workers are identical and 100% reliable.
- No preemptions occur in scheduling.
- The optimal schedule is based on a single unloading activity. Thus, the picking order of each prescription proceeds once the medicine pallets are completely unloaded.

In the stochastic model formulation, the processing time for breaking down the medicine pallets and picking order of the prescription was assumed to be random variables subjected to a normal distribution, while the due date of the picking order was assumed to be a random variable subject to a uniform distribution.

### 3.1. Indices

 
*i,i'*: incoming medicine pallet (*i,i'* = 1,…,*n*)
 
*j,j'*: outgoing medicine container (*j*, *j'* = 1,…,*o*)
 
*k*: worker (*k* = 1,…,*m*)
 
*l*: transfer equipment; TF (*l* = 1,…,*q*)

### 3.2. Decision Variables

 
*x*_*ik*_ is 1 if worker *k* is assigne d for incoming pallet *i*;  otherwise, 0
 
*y*_*jk*_ is 1 if worker *k* is assigne d for outgoing container *j*;  otherwise, 0
 
*I*_*ii*′*k*_ is 1 if incoming pallet *i* prece de s *i*′ by the same worker *k*;  otherwise, 0
 
*J*_*jj*′*k*_ is 1 if outgoing container *j* prece de s *j*′ by the same worker *k*;  otherwise, 0
 
*a*_*ii*′*l*_ is 1 if incoming pallet *i* prece de s *i*′ by the same TF *l*;  otherwise, 0
 
*b*_*jj*′*l*_ is 1 if outgoing container *j* prece de s *j*′ by the same TF *l*;  otherwise, 0
 
*U*_*ikl*_ is 1 if worker *k* an d tool *l* are assigne d for incoming pallet *i*;  otherwise, 0
 
*V*_*jkl*_ is 1 if outgoing container *j* is processe d by worker *k* an d TF *l*;  otherwise, 0
 
*α*_*ikl*_ is the start time of medicine incoming pallet *i* break down by worker *k* TF *l*
 
*β*_jkl_ is the start time of outgoing medicine container *j* preparation by worker *k* TF *l*
 
*c*_ikl_ is the completion time of incoming pallet *i* break down by team *k* TF *l*
 
*z*_jkl_ is the completion time of outgoing medicine container *j* by worker *k* TF *l*
 
*t*_*j*_ is the tardiness of outgoing medicine container *j*

### 3.3. Parameters

 
*n* is the number of incoming medicine pallets
 
*o* is the number of outgoing medicine containers
 
*m* is the number of workers
 
*q* is the number of transfer equipment
 
*r*_*i*_ is the ready time of incoming medicine pallet *i* break down
 
*p*MU_*i*_ is the mean processing time required to break down incoming medicine pallet *i*
 
*p*STD_*i*_ is the standard deviation of processing time required to break down incoming medicine pallet *i*
 
*po*MU_*j*_ is the mean processing time for preparing outgoing medicine container *j*
 
*po*STD_*j*_ is the standard deviation of processing time for preparing outgoing medicine container *j*
 
*dU*_*j*_ is the upper point of due date of outgoing medicine container *j*
 
*dL*_*j*_ is the lower point of due date of outgoing medicine container *j*
 
*S*_*ij*_ is 1 if outgoing medicine container *j* is pulled from medicine pallet *i*; otherwise, 0
 
*h*_*il*_ is 1 if incoming medicine pallet *i* can be transferred by TF *l*; otherwise, 0
 
*f*_*jl*_ is 1 if outgoing medicine container *j* can be transferred by TF *l*; otherwise, 0
 
*G* is a large number
 
*ρ* is the confidence level for satisfaction with the constraint set, 1 ≥ *ρ* ≥ 0

### 3.4. Objective Function

(1) $$\begin{array}{c} \begin{array}{cc} Minimise & \overset{n}{\underset{j=1}{\sum }}t_{j},\forall j \end{array}\text{ .} \end{array}$$

Equation (1) is the objective function for minimising the total tardiness.

### 3.5. Constraints

Equations (2) and (3) ensure that each incoming medicine pallet and outgoing medicine container must be processed by one worker, respectively.

(2) $$\begin{array}{c} \overset{m}{\underset{k=1}{\sum }}x_{ik}=1\;,\forall i\text{,} \end{array}$$

(3) $$\begin{array}{c} \overset{m}{\underset{k=1}{\sum }}y_{jk}=1,\forall j\text{.} \end{array}$$

Equations (4)–(7) guarantee the precedence relationship when incoming medicine pallets or outgoing medicine containers are processed with the same worker [11]:

(4) $$\begin{array}{c} x_{ik}+x_{i^{\prime }k}-\left(I_{ii^{\prime }k}+I_{i^{\prime }ik}\right)\leq 1,\forall ii^{\prime }k,i\neq i^{\prime }\text{,} \end{array}$$

(5) $$\begin{array}{c} 2\left(I_{ii^{\prime }k}+I_{i^{\prime }ik}\right){-x}_{ik}-x_{i^{\prime }k}\leq 0,\forall ii^{\prime }k,i\neq i^{\prime }\text{,} \end{array}$$

(6) $$\begin{array}{c} y_{jk}+y_{j^{\prime }k}-\left(J_{jj^{\prime }k}+j_{j^{\prime }jk}\right)\leq 1,\forall jj^{\prime }k\;,j\neq j^{\prime }\text{,} \end{array}$$

(7) $$\begin{array}{c} 2\left(J_{jj^{\prime }k}+J_{j^{\prime }jk}\right){-y}_{jk}-y_{j^{\prime }k}\leq 0,\forall jj^{\prime }k\;,j\neq j^{\prime }\text{.} \end{array}$$

Equations (8) and (9) guarantee the relationships when incoming medicine pallets or outgoing medicine containers are transferred by the same transfer equipment, respectively.

(8) $$\begin{array}{c} U_{ikl}+U_{i^{\prime }kl}-\left(a_{ii^{\prime }l}+a_{i^{\prime }il}\right)\leq 1\;,\forall ii^{\prime }l\;,i\neq i^{\prime }\text{,} \end{array}$$

(9) $$\begin{array}{c} V_{jkl}+V_{j^{\prime }kl}-\left(b_{jj^{\prime }l}+b_{j^{\prime }jl}\right)\leq 1,\forall jj^{\prime }l\;,j\neq j^{\prime }\text{.} \end{array}$$

Equations (10)–(13) ensure that each incoming medicine pallet or outgoing medicine container must be transferred by only one transfer equipment that is eligible for use.

(10) $$\begin{array}{c} \overset{m}{\underset{k=1}{\sum }}\overset{q}{\underset{l=1}{\sum }}U_{ikl}=1\;,\forall i\text{,} \end{array}$$

(11) $$\begin{array}{c} \overset{m}{\underset{k=1}{\sum }}\overset{q}{\underset{l=1}{\sum }}V_{jkl}=1,\forall j\text{,} \end{array}$$

(12) $$\begin{array}{c} \overset{m}{\underset{k=1}{\sum }}\overset{q}{\underset{l=1}{\sum }}U_{ikl}\times h_{il}\times x_{ik}=1\;,\forall i\text{,} \end{array}$$

(13) $$\begin{array}{c} \overset{m}{\underset{k=1}{\sum }}\overset{q}{\underset{l=1}{\sum }}V_{jkl}\times f_{jl}\times y_{ik}=1\;,\forall j\text{.} \end{array}$$

Equations (14)–(17) state that the probability of the constraint sets must be satisfied by at least *ρ* to ensure sufficient time between breaking down an incoming medicine pallet and to prepare an outgoing medicine container for each worker or piece of transferring equipment.

(14) $$\begin{array}{c} Prob\left\{\left.c_{ikl}\leq \left(c_{i^{\prime kl}}-p_{i^{\prime }}\right)+G\left(1-I_{ii^{\prime }k}\right)\right\}\geq \rho \right.,\forall l,\forall ii^{\prime }k\;,i\neq i^{\prime }\text{,} \end{array}$$

(15) $$\begin{array}{c} Prob\left\{c_{ikl}\leq \left(c_{i^{\prime kl}}-p_{i^{\prime }}\right)+G\left(1-a_{ii^{\prime }l}\right)\right\}\geq \rho ,\forall k\;,\forall ii^{\prime }l\;,i\neq i^{\prime }\text{,} \end{array}$$

(16) $$\begin{array}{c} Prob\left\{z_{jkl}\leq \left(z_{j^{\prime }kl}-{po}_{j^{\prime }}\right)+G\left(1-J_{jj^{\prime }k}\right)\right\}\geq \rho ,\forall ljj^{\prime }k\;,j\neq j^{\prime }\text{,} \end{array}$$

(17) $$\begin{array}{c} Prob\left\{\left.z_{jkl}\leq \left(z_{j^{\prime }kl}-{po}_{j^{\prime }}\right)+G\left(1-b_{jj^{\prime }l}\right)\right\}\geq \rho \right.,\forall kjj^{\prime }l\;,j\neq j^{\prime }\text{.} \end{array}$$

Equations (18) and (19) enforce the start time of breaking down an incoming medicine pallet and start time of preparing an outgoing medicine container, respectively.

(18) $$\begin{array}{c} \alpha_{ikl}\geq r_{i\;},\forall ikl\text{,} \end{array}$$

(19) $$\begin{array}{c} \beta_{jkl}\geq c_{ikl},\forall ijkl\text{.} \end{array}$$

Equations (20) and (21) state that the probability of the constraint sets must be satisfied by at least *ρ* to ensure the ready times of braking down an incoming medicine pallet and preparing an outgoing medicine container, respectively.

(20) $$\begin{array}{c} Prob\left\{\left.c_{ikl}-r_{i}\geq p_{i\;}\right\}\geq \rho \right.,\forall ikl\text{,} \end{array}$$

(21) $$\begin{array}{c} Prob\left\{\left.z_{jkl}-c_{ikl}\geq {po}_{j}\right\}\geq \rho \right.\;,\forall jk;\;i=1st\;,..,last\;predecessor\;of\;\;j\text{.} \end{array}$$

Equations (22) and (23) state that the probability of the constraint sets must be satisfied by at least *ρ* to determine the completion time of each incoming medicine pallet and outgoing medicine container, respectively.

(22) $$\begin{array}{c} Prob\left\{\alpha_{ikl}+p_{i}\geq c_{i\;}\right\}\geq \rho ,\forall ikl\text{,} \end{array}$$

(23) $$\begin{array}{c} Prob\left\{\beta_{jkl}+{po}_{j}\geq z_{j\;}\right\}\geq \rho ,\forall ikl\text{.} \end{array}$$

Equation (24) ensures that the probability of tardiness for outgoing medicine container *j* must be satisfied by at least *ρ*.

(24) $$\begin{array}{c} Prob\left\{t_{j}\geq \max\;\left(0,c_{jkv}-d_{j}\right)\right\}\geq \rho ,\forall j,k\;,v\text{.} \end{array}$$

Equation (25) specifies that all decision variables are binary.

(25) $$\begin{array}{c} y_{ik},Y_{jk},I_{ii^{\prime }k},J_{jj^{\prime }k},a_{ii^{\prime }l},A_{jj^{\prime }l},U_{ikl},V_{jkl}\in \left\{0,1\right\}\text{.} \end{array}$$

## 4. Classical Metaheuristic Approach

### 4.1. GA

The genetic algorithm (GA) is an adaptive search technique used to solve optimisation problems. Although the GA was developed much earlier than 1975, the basic principles of GA were first emphasized by Holland in 1975 [50]. The GA evolution procedure is based on the laws of natural selection and genetics. In the GA, the solutions are represented as chromosomes. The chromosomes are evaluated for fitness values and ranked from best to worst based on their fitness values.

During a genetic operation, chromosomes are selected from the population and recombined to produce offspring that comprise the population of the next generation. This process is accomplished through repeated applications of three genetic operators: selection, crossover, and mutation.

### 4.2. PSO

Particle swarm optimisation (PSO) is a population-based random search method that imitates the physical movements of individuals in a swarm as a searching mechanism. The PSO algorithm was originally proposed by Eberhart and Kennedy in 1995 [51]. Similar to the GA, the population in PSO is initialised with random solutions. In PSO, a solution is represented as a particle, and the population of solutions is called a swarm of particles. Each particle has two main attributes: position and velocity. The difference between PSO and GA lies in evolutionary procedures. The key concept of PSO is that each particle learns from the cognitive knowledge of its experiences (personal best, *pbest*) and the social knowledge of the swarm (global best, *gbest*) to guide itself to a better position. A particle moves to a new position using an updated velocity. Once a new position is reached, the best position of each particle and the best position of the swarm are updated as needed. The velocity of each particle is then adjusted based on the particle experiences. This process is repeated until a stopping criterion is satisfied.

### 4.3. DE

Differential evolution (DE), introduced by Storn and Price in 1995 [13], is an evolutionary algorithm designed to deal with continuous optimisation problems. Similar to GA and PSO, DE is a population-based random search algorithm. In DE, the initial *D*-dimensional vector population of size *N* is randomly generated and should cover the entire search space. Typically, the DE population evolves through repeated cycles of three main DE operators: mutation, crossover, and selection. However, DE has a different mechanism for generating new solutions explained as follows.

#### 4.3.1. Mutation

A mutant vector is generated by combining three vectors randomly selected from the population, excluding the target vector, *X*_*G*_. Equation (26) shows the combination process of three randomly selected vectors to form the mutant vector *V*_*G*_ as follows:

(26) $$\begin{array}{c} V_{G}=X_{1,G}+F\left(X_{2,G}–X_{3,G}\right)\text{,} \end{array}$$

where *X*_1*,G*_, *X*_2*,G*_, and *X*_3*,G*_ are three randomly selected vectors from the population in generation *G* and *F* is a scale factor which is the main parameter of the DE algorithm.

#### 4.3.2. Crossover

To increase the diversity of the perturbed parameter vectors after mutation, a trial vector, *U*_*G*_, is generated by a crossover operation. The crossover probability (0 ≤ *C*_*r*_ ≤ 1) must be specified for the crossover operator to control the probability of selecting the value in each dimension from a mutant vector. In classic DE, the uniform crossover is employed, and the trial vector is generated using the following equation:

(27) $$\begin{array}{c} U_{j,G}=\left\{\begin{array}{cc} V_{j,G}\text{,} & if\;\left(r\right.andb\left(j\right)\leq C_{r}\text{,}\\ X_{j,G}\text{,} & Otherwise\text{,} \end{array}\right. \end{array}$$

where *randb(j)* is the *j*^*th*^ evaluation of a uniform random number generator with the outcome ∈ [0, 1].

#### 4.3.3. Selection

To determine a survival vector for the next generation, a comparison of fitness values between a trial vector, *U*_*G*_, and a target vector, *X*_*G*_, is performed in the selection operator. The simple criterion is to maintain the vector with a better fitness value. If *U*_*G*_ yields a better fitness value than *X*_*G*_, *X*_*G + 1*_ is set to *U*_*G*_; otherwise, the old value *X*_*G*_ is retained.

### 4.4. RAM-EPSDE

Ranked-Based Mutation Adaptation (RAM), proposed by Leon and Xiong in 2018, is a selection method of different mutation strategies of DE. In RAM, the mutation strategy was selected according to probabilities of each population subgroups. RAM was used to enhance the classical DE algorithm by integrating with other adaptive DE algorithms.

Ensemble of Parameters and Mutation Strategies DE (EPSDE) algorithm, proposed by [31], was another adaptive parameter and searching strategies DE. EPSDE randomly selected different mutation and crossover strategies and parameter values in its pool listed as follows:

1. Mutation strategies are DE/best/2/bin, DE/rand/1, and DE/current-to-rand/1/bin
2. Crossover strategies are binomial crossover and exponential crossover
3. Parameter values are the population size (*NP* = 50), *F* ∈ [0.5, 0.9], and CR ∈ [0.1, 0.5, 0.9]

Later, Leon et al. [19] introduced an integration of RAM into EPSDE and named the algorithm as RAM-EPSDE with an aim to improve the search efficiency.

## 5. SSDE

### 5.1. SSDE Framework

Selective strategy DE (SSDE) is proposed in this study to enhance the DE performance. The modifications to the classical DE algorithm are listed in Table 1. In contrast to the classical DE, SSDE uses six different strategies obtaining from the combination of mutation and crossover schemes proposed by Price et al. [29] in order to maintain both the exploitation and exploration abilities of DE [29]. In the classic DE, a new population is generated using one strategy, while a new population of SSDE can be generated using various strategies. The ability to adjust itself to use different potential strategies towards a better solution is the key to enhancing the search speed of the SSDE.

In addition, to improve the computational time cost, other termination conditions are added to SSDE as follows:

- Maximum iterations: similar to classical DE and other evolutionary algorithms, SSDE algorithm is terminated when the maximum number of iterations is reached
- No further improvement: different from the termination condition of classical DE, the algorithm is terminated when an improvement probability does not increase for a specific number of *n* iterations
- The optimal solution is obtained: when the objective reaches the target value, for example, zero tardiness, the algorithm is terminated

### 5.2. SSDE Procedure

**Figure 2:**
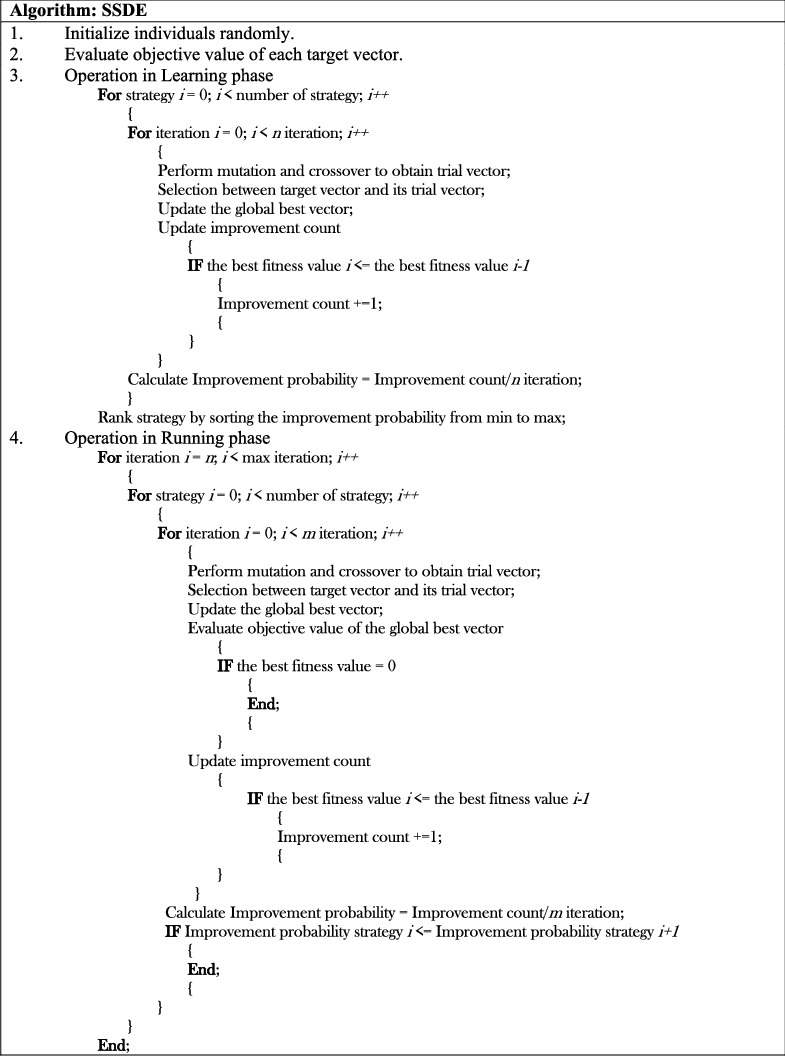
The pseudocode of the SSDE algorithm.

The SSDE procedure is divided into two main stages: learning and running. In the learning stage, each strategy is used to perform the evaluation process for *n* iterations. Then, the ability to achieve a better fitness value for each strategy is recorded according to the improvement probability. Next, the improvement probabilities of all strategies are sorted in ascending order to rank for qualities. In the running stage, the improvement probability is updated for every *m* iterations. First, the evolutionary process is performed by employing the first-rank strategy. Subsequently, if the improvement probability of the candidate strategy is lower than that of the others after the update, SSDE switches its strategy to the highest improvement probability strategy. This process is repeated until the algorithm terminates. The pseudocode of the SSDE algorithm with the proposed modification is shown in Figure 2.

## 6. Solution Representation

Since evolutionary algorithms were developed for continuous optimisation, each individual in the population must be transformed into a practical solution. In this study, a solution representation with encoding and decoding was developed to obtain the schedule of internal tasks in a cross-dock terminal with the minimum total tardiness.

**Figure 3:**
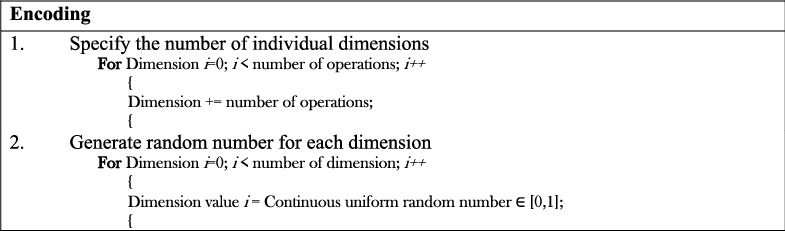
The pseudocode of the encoding procedure.

In the encoding procedure, the number of dimensions was set to be equal to the total number of operations in a single unloading activity. Then, a uniform random number was generated for each dimension in the interval [0, 1]. The pseudocode of the encoding procedure is shown in Figure 3.

**Figure 4:**
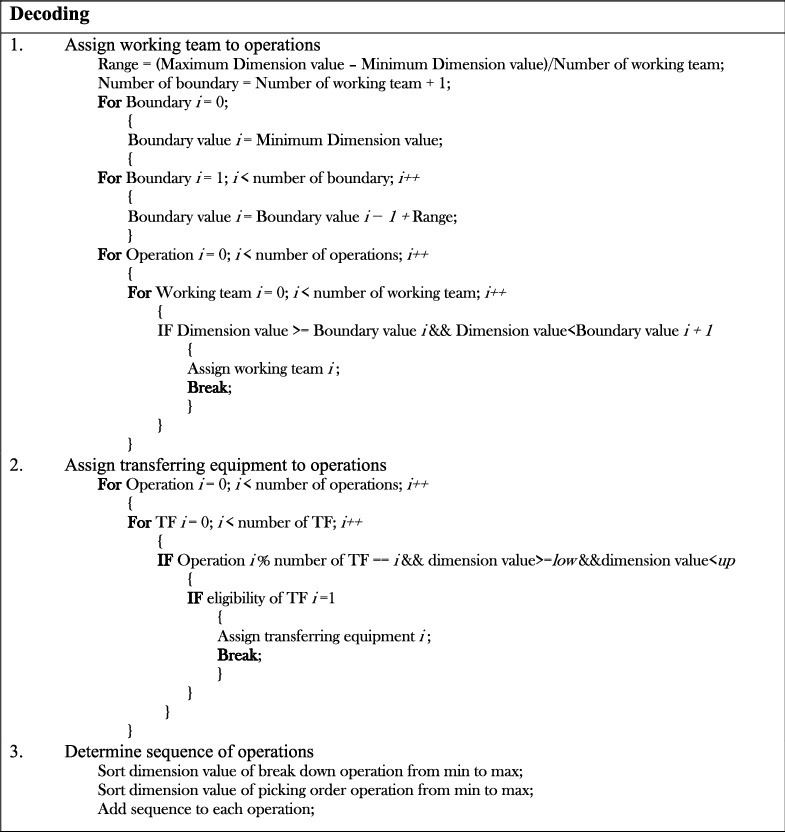
Pseudocode of the decoding procedure.

The decoding procedure transforms random numbers of each dimension into a practical solution. The decoding procedure consists of the following steps:

  Step 1: defining the condition for assigning workers to operations according to the range and the boundary value, which are calculated from the dimension value and number of workers.
  Step 2: allocating the transferring equipment to operations in a balanced manner. The assignment in this step is based on the dimension value and its eligibility for handling that operation.
  Step 3: applying a sorting list rule to generate a sequence of operations.
  Step 4: generating random values of processing times and due dates according to the stochastic environment of the problem. Then, the start time, end time, the tardiness of each operation, and the total tardiness were calculated according to the random processing times and due dates. The pseudocode of the decoding procedure is shown in Figure 4.

## 7. Computational Experiments

The computational experiments were divided into two parts. SSDE performance is first tested using benchmark functions. Then, SSDE is used for solving the larger-scale problem of internal tasks scheduling in a cross-docking terminal. The performance of SSDE was evaluated and compared with other metaheuristics, GA, PSO, DE, and RAM-EPSDE. The analysis of numerical results in terms of solution quality and convergence behaviour was also investigated. It is noted that the computational experiments were run on an Intel® Pentium IntelCoreTM i5 8^th^ Gen CPU (1.60 GHz) with 8 GB RAM.

### 7.1. Benchmark Test Functions

In this section, the performance of the SSDE is verified using five benchmark functions. These functions are bound-constrained high-dimensional single-objective optimisation (Dim = 100/1000/2000) of CEC 2020 [52]. The unimodal functions *F*1 and *F*2 have only one global optimal value, so they can be used to test the local search ability of the algorithm, while the multimodal functions *F*3–*F*5 with more than two optimal values are used to evaluate the global exploration ability. The benchmark functions used in this study are listed in Table 2.

The numeric experiment results obtained from SSDE were compared with those from other algorithms, including MFO [53], SOGWO [54], PFA [55], EO [56], and MMPA [57]. The parameter setting of the comparison algorithms is shown in Table 3.

These parameters were selected according to the suggestions presented by the developers of the algorithms in the aforementioned references. The population size of each algorithm was set to 30, and the number of iterations was set to 500.  Table 4 shows the comparison results of SSDE and other existing algorithms obtained from 30 independent runs.

According to the results in Table 4, although the SSDE is not able to obtain better solutions than SOGWO and MMPA, it performs better than PSO, MFO, and PFA in both unimodal and multimodal functions. This can be due to the fact that SSDE was particularly developed for solving a combinatorial problem, not a continuous optimisation problem. In the next section, experiments are conducted to demonstrate the performance of SSDE in solving internal tasks scheduling problems in cross-docking terminals.

### 7.2. Parameter Setting and Instances of Internal Task Scheduling Problem

To determine the best set of key parameter values of each algorithm, the Taguchi method was used to reduce the computational load from a full factorial experiment. For each algorithm, a three-factor with four-level experiment was performed on three instances of the scenario of the real-world cross-docking problem. It is noted that, in this study, the parameters of the RAM-EPSDE were set according to the original EPSDE in [31]. In addition, the number of function evaluations (number of iterations × population size) was set to a fixed value of 50,000 for all algorithms. The best parameter values for each algorithm derived from the parameter settings are listed in Table 5.

The computational experiment was performed on 15 instances. Instances 1 to 7 were small-size problems with generated data. Instances 8 to 15 were large-size problems in which the data were derived from the real-scenario of a medicine distribution centre. The problem size is identified by four generated parameters which are number of workers (*m*), number of transfer equipment (*q*), number of incoming medicine pallets (*n*), and number of outgoing medicine containers (*o*). In addition, to make the problem more practical, in some instances, some customer orders are restricted for the use of particular transferring equipment.

### 7.3. Experimental Results on the Instances of Internal Task Scheduling Problem

In this section, the performance of the proposed SSDE was evaluated using the real-case scenarios of a pharmaceutical distribution centre. The experimental results obtained from the SSDE, in terms of solution quality and computational time, were compared to solutions obtained from the optimisation solver LINGO and other metaheuristics. It is noted that the comparison of all metaheuristics was executed under the same conditions of solution representation and total number of function evaluations.  Table 6 shows the comparison results of total tardiness among different metaheuristics. The best, average, and standard deviation of total tardiness obtained from those algorithms are also reported.

It can be seen from Table 6 that, for small-size problems, the total tardiness obtained from the metaheuristic method was lower than the exact method. This may be because of a stochastic setting in the mathematical model of the problem. In addition, there was no difference in the recording results including the best, mean, and standard deviation among metaheuristic methods. However, for large-sized problems, LINGO could not find solution since it ran out of memory for generating the model. Among metaheuristics, SSDE generally provided superior solution quality than other algorithms since most of the best, average, and standard deviation values of total tardiness are lower. In addition, SSDE clearly outperformed GA, PSO, classic DE, and RAM-EPSDE by providing solutions with zero tardiness values and zero standard deviations for most instances. It is noteworthy that the quality of the solutions obtained by the metaheuristic methods deteriorated as the problem size increased. This may suggest that newly generated solutions could not escape from the local optimal when the problem became more complex. Therefore, a high diversification for generating new populations leads to a better solution. Consequently, GA provided the highest total tardiness, whereas PSO, classic DE, and RAM-EPSDE yielded lower total tardiness. In conclusion, the numerical results showed that SSDE yielded the best total tardiness and computational time.

Moreover, a one-sided *t*-test was performed to compare SSDE performance with those of the classical DE algorithm and RAM-EPSDE in terms of the average total tardiness for large-size instances. The *t*-value according to equation (28) was calculated to confirm the significant difference of compared algorithms.

(28) $$\begin{array}{c} t=\frac{\left({\bar{x}}_{Compared\;algo}-{\bar{x}}_{SSDE}\right)}{\sqrt{s_{Compared\;algo}^{2}/n_{Compared\;algo}+s_{SSDE}^{2}/n_{SSDE}}}\text{.} \end{array}$$

In Equation (28), $\bar{x}$ and *s* are the mean and standard deviations of total tardiness obtained from 30 replicated runs (*n*), respectively. It is noteworthy that *α* is an uncertainty level, set at 5% for this experiment, and *n* is the degree of freedom (*n* = 30 − 1 = 29). Hence, the critical value of *t*_0.05, 29_ is equal to 1.699. If *t*-value is higher than the critical value, the average total tardiness obtained from SSDE is significantly lower.  Table 7 shows the results of a one-sided *t*-test of average total tardiness. It can be seen that the *t*-values of average total tardiness were higher than the critical value for all large-size instances. Thus, it was clear that the total tardiness obtained from the SSDE was significantly lower than that of the classical DE and RAM-EPSDE with 95% confidence level.

**Figure 5:**
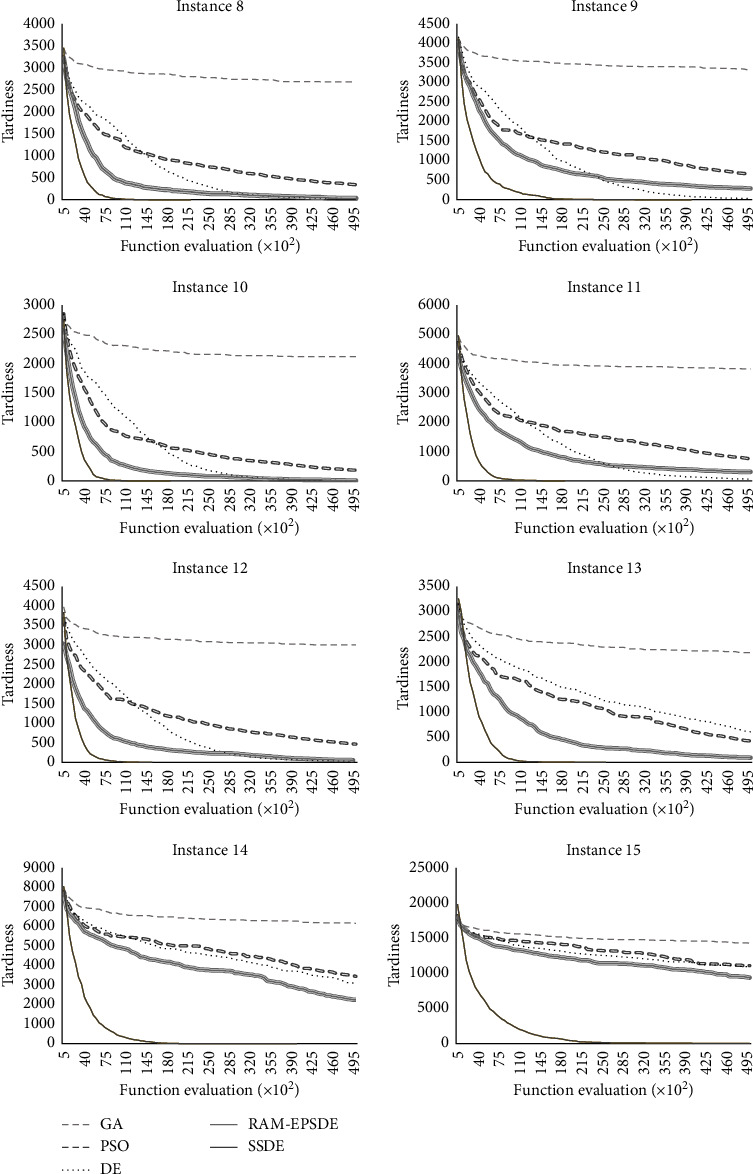
Comparison of convergence behaviour among algorithms.

In addition, the ability to escape from the local optimal and attain a better solution can be observed by behaviour of convergence. In this study, the comparison of convergence behaviour among different algorithms was investigated using the graph of average fitness values versus the number of function evaluations as shown in Figure 5.

According to Figure 5, GA showed the lowest convergence speed, while PSO and DE yielded better speeds. This may be because the task scheduling problem requires diversification to generate new populations through an evolutionary process and handle a large number of prescriptions. The convergence speeds of RAM-EPSDE were better than the classical DE due to the use of multisearching strategies. Although integrating RAM with EPSDE leads to an improvement of the original DE, crossover strategies and parameter settings are not considered in the adaptation. In contrast, SLDE adapts both of its mutation and crossover strategies. In addition, the parameter values of SLDE were derived from the parameter testing using the real-case study. Consequently, SLDE showed its robustness compared to RAM-EPSDE in internal task scheduling problems. In addition, SSDE showed its outstanding competence in escaping from the local optimal and reaching a better solution. The superior performance of the SSDE is attributed to the application of various modified DE strategies and self-adaptive methods through the evolutionary processes of SSDE.

## 8. Conclusion

A novel SSDE algorithm was proposed to handle the complexity of stochastic internal task scheduling problems in cross-dock terminals. The aim of this study was to assign workers and transfer equipment to operations and to sequence those operations to minimise total tardiness. The ability to adapt itself to execute the best strategy was the key to the success of SSDE. In SSDE, fitness values were considered to prioritise the capability of each strategy in the learning stage. Subsequently, the improvement probability was used in the strategy selection during the running stage.

In this study, a solution representation with encoding and decoding procedures was also developed to transform a random number into a scheduling solution. Then, an exact method using the LINGO optimisation solver and metaheuristics was applied to solve 15 instance problems using generated data based on a real-case scenario of a pharmaceutical distribution centre. The performance of the SSDE was compared with the results obtained from LINGO and other classical metaheuristics in terms of solution quality and computational time. Based on the experimental results, there was no difference in the solution quality when solving small problems. However, LINGO took a long time to solve small problems and suffered from insufficient working memory when attempting to solve large problems. In contrast, all metaheuristic algorithms required less computing time to provide solutions for all instances. Based on numerical results and convergence behaviour, SSDE significantly outperformed classical DE and other algorithms in terms of both solution quality and computational time. In addition, SSDE presented faster convergence than the other algorithms. Hence, SSDE is an alternative approach for solving stochastic internal task scheduling problems in cross-dock terminals.

## Figures and Tables

**Table 1:** Modifications in DE algorithm.

No.	Modification	Classical DE	SSDE
1	Mutation operator	1 scheme *V*_*G*_ *=* *X*_1*,G*_ *+* *F(X*_1*,G*_ − *X*_2*,G*_)	3 schemes *V*_*G*_ *=* *X*_1*,G*_ *+* *F(X*_1*,G*_ − *X*_2*,G*_) *V*_*G*_ *=* *X*_*b*_,_*G*_ *+* *F(X*_2*,G*_ − *X*_2*,G*_) *V*_*G*_ *=* *X*_*b*_,_*G*_ *+* *F(X*_1*,G*_*– X*_2*,G*_) *+* *F(X*_3*,G*_ − *X*_4*,G*_)
2	Crossover operator	1 scheme *Binomial crossover*	2 schemes *Binomial crossover* *Exponential crossover*
3	Generating new population	Using a DE strategy in a single run	Using various DE strategies in a single run *There are 6 strategies from the combination of mutation and crossover schemes*
4	Termination condition	1 condition *Max iterations*	3 condition *Max iterations* *No more improvement* *Objective reach to the target*

**Table 2:** The benchmark functions.

ID	Function	Range	*f*_min_
*F*1	*f*(*x*)=∑_*i*=1_^*n*^|*x*_*i*_|+∏_*i*=1_^*n*^|*x*_*i*_|	[−10, 10]	0
*F*2	*f*(*x*)=∑_*i*=1_^*n*^(*x*_*i*_+0.5)^2^	[−100, 100]	0
*F*3	(*x*)=∑_*i*=1_^*n*^[*x*_*i*_^2^ − 10 cos (2*πx*_*i*_)+10]	[−5.12, 5.12]	0
*F*4	$f\left(x\right)=-20\;\exp\;\left(-20\sqrt{1/n\sum_{i=1}^{n}x_{i}^{2}}-\exp\;\left(1/n\sum_{i=1}^{n}\cos\;\left(2\pi x_{i}\right)\right)\right.$	[−32, 32]	0
*F*5	$f\left(x\right)=1/4000\sum_{i=1}^{n}x_{i}^{2}-\prod_{i=1}^{n}\cos\left(x_{i}/\sqrt{i}\right)+1$	[−600, 600]	0

**Table 3:** Parameter setting for testing the benchmark functions.

Algorithm	Parameters
MFO	*a* = −1 (linearly decreased over iterations)
SOGWO	*a* = 2 (linearly decreased over iterations)
PFA	∼
EO	*a*1 = 2, *a*2 = 1, *GP* = 0.5, *t* = 1 (nonlinearly decreased over iterations)
MMPA	*FADs* = 0.2
SSDE	*F* = 0.2, *Cr* = 0.9

**Table 4:** Comparison results among different algorithms for unimodal and multimodal functions with 100 D/1000 D/2000 D.

ID	Dimensions	PSO		MFO		PFA		SOGWO		MMPA		SSDE		
		Mean	Std	Mean	Std	Mean	Std	Mean	Std	Mean	Std	Mean	Std	Best
*F*1	100	3.87*E* + 01	9.22*E* + 00	2.42*E* + 02	4.33*E* + 01	2.51*E* − 17	7.89*E* − 02	4.16*E* − 08	1.45*E* − 08	0.00*E* + 00	0.00*E* + 00	6.70*E* + 01	4.39*E* + 00	5.17*E* + 01
	1000	1.39*E* + 03	5.48*E* + 01	Infeasible		Infeasible		6.62*E* − 01	3.00*E* − 01	0.00*E* + 00	0.00*E* + 00	1.28*E* + 03	6.01*E* + 01	1.13*E* + 03
	2000	1.52*E* + 42	7.71*E* + 42	Infeasible		Infeasible		9.74*E* + 00	3.79*E* + 00	3.47*E* − 51	1.32*E* − 50	2.59*E* + 04	1.58*E* + 03	2.26*E* + 04

*F*2	100	2.02*E* + 01	5.55*E* + 00	5.83*E* + 04	1.31*E* + 04	4.36*E* + 00	1.63*E* + 00	1.01*E* + 01	1.10*E* + 00	4.41*E* + 00	4.17*E* − 01	9.81*E* + 01	6.40*E* + 00	6.76*E* + 01
	1000	4.10*E* + 04	1.86*E* + 03	1.30*E* + 04	4.94*E* + 04	2.29*E* + 05	2.74*E* + 04	2.03*E* + 02	2.03*E* + 02	1.94*E* + 02	3.20*E* + 00	3.63*E* + 04	3.08*E* + 03	3.10*E* + 04
	2000	1.88*E* + 05	6.32*E* + 03	5.94*E* + 06	6.94*E* + 04	1.02*E* + 06	9.48*E* + 04	8.23*E* + 04	8.00*E* + 04	4.26*E* + 02	2.52*E* + 00	7.56*E* + 05	7.87*E* + 04	5.86*E* + 05

*F*3	100	6.06*E* + 02	7.36*E* + 01	8.59*E* + 02	7.15*E* + 01	4.87*E* + 02	6.42*E* + 01	7.63*E* + 00	5.54*E* + 00	0.00*E* + 00	0.00*E* + 00	2.62*E* + 02	2.94*E* + 01	1.98*E* + 01
	1000	1.47*E* + 04	6.48*E* + 02	1.55*E* + 04	2.09*E* + 02	1.03*E* + 04	4.34*E* + 02	1.99*E* + 02	4.99*E* + 01	0.00*E* + 00	0.00*E* + 00	7.31*E* + 03	4.01*E* + 02	6.26*E* + 03
	2000	3.15*E* + 04	1.03*E* + 03	3.31*E* + 04	2.74*E* + 02	2.35*E* + 04	8.57*E* + 02	5.59*E* + 02	1.07*E* + 02	0.00*E* + 00	0.00*E* + 00	1.92*E* + 04	6.38*E* + 02	1.72*E* + 04

*F*4	100	3.73*E* + 00	2.96*E* − 01	1.99*E* + 01	9.58*E* − 02	6.18*E* + 00	6.46*E* + 00	1.44*E* − 07	5.68*E* − 08	8.88*E* − 16	0.00*E* + 00	5.41*E* + 00	7.62*E* − 01	3.76*E* + 00
	1000	1.59*E* + 01	2.90*E* − 01	2.04*E* + 01	2.03*E* − 01	1.99*E* + 01	5.90*E* − 01	1.92*E* − 02	2.83*E* − 03	8.88*E* − 16	0.00*E* + 00	8.23*E* + 00	5.05*E* − 01	6.86*E* + 00
	2000	1.76*E* + 01	1.13*E* − 01	2.04*E* + 01	2.67*E* − 01	2.04*E* + 01	2.99*E* − 01	1.18*E* − 01	1.50*E* − 02	8.88*E* − 16	0.00*E* + 00	9.86*E* + 00	4.37*E* − 01	8.75*E* + 00

*F*5	100	3.93*E* − 01	9.30*E* − 02	5.30*E* + 02	1.62*E* + 02	6.93*E* − 01	2.20*E* − 01	8.69*E* − 03	1.25*E* − 02	0.00*E* + 00	0.00*E* + 00	6.16*E* + 00	2.23*E* + 00	1.99*E* + 00
	1000	2.80*E* + 02	1.71*E* + 01	2.45*E* + 04	5.24*E* + 02	2.16*E* + 03	2.18*E* + 02	1.55*E* − 01	1.16*E* − 01	0.00*E* + 00	0.00*E* + 00	2.84*E* + 02	3.26*E* + 01	2.13*E* + 02
	2000	7.55*E* + 02	3.21*E* + 01	5.36*E* + 04	6.40*E* + 02	9.22*E* + 03	8.38*E* + 02	1.14*E* + 00	2.22*E* − 01	0.00*E* + 00	0.00*E* + 00	7.62*E* + 02	1.42*E* + 02	4.57*E* + 02

**Table 5:** Best parameter values for each algorithm.

Algorithm	Parameters and their best level		
	Function evaluation	Mutation rate	Crossover rate, *C*_*r*_
GA	100 × 500	0.10	0.3
	Function evaluation	Inertia weight	Acceleration (*C*_*p*_, *C*_*g*_)
PSO	200 × 250	0.6–1.0	0.7, 0.7
	Function evaluation	Scale factor, *F*	Crossover rate, *C*_*r*_
DE	500 × 100	1.0	0.6
RAM-ESPDE	1000 × 50	[0.5, 0.9]	[0.1, 0.5, 0.9]
SSDE	100 × 500	2.0	0.9

**Table 6:** Comparison of total tardiness and computational time among different algorithms.

Ins	Problem size	Lingo		GA				PSO				DE				RAM-EPSDE				SSDE			
	(*m*, *q*, *n*, *o*)	Tardiness	Run time (s)	Tardiness			Run time (s)	Tardiness			Run time (s)	Tardiness			Run time (s)	Tardiness			Run time (s)	Tardiness			Run time (s)
				Best	Avg	SD	Avg	Best	Avg	SD	Avg	Best	Avg	SD	Avg	Best	Avg	SD	Avg	Best	Avg	SD	Avg
1	2, 2, 3, 3	0.97	35.2	1.0	1.4	0.4	0.1	1.0	1.4	0.3	0.2	1.1	1.6	0.5	0.1	1.0	1.3	0.3	0.2	1.0	1.5	0.4	0.2
2	2, 2, 4, 3	9.15	137.4	6.9	8.5	0.8	0.2	6.3	8.2	1.0	0.2	6.2	8.5	1.3	0.2	9.2	10.0	1.0	0.3	6.8	8.9	1.0	0.3
3	2, 2, 4, 4	12.12	513.5	12.6	14.0	0.7	0.2	12.9	13.9	0.6	0.2	12.5	14.0	0.5	0.2	13.2	14.0	0.4	0.3	12.7	14.0	0.5	0.3
4	3, 3, 4, 5	30.31	1169.2	9.5	10.5	0.6	0.2	9.0	10.7	0.7	0.3	9.2	10.4	0.6	0.2	9.4	10.3	0.6	0.3	9.3	10.4	0.6	0.3
5	3, 3, 4, 6	29.39	1452.1	8.4	9.5	0.5	0.3	8.5	9.5	0.5	0.3	8.8	9.5	0.4	0.3	9.1	9.7	0.5	0.3	8.5	9.6	0.5	0.3
6	3, 3, 4, 7	72.63	15579.7	18.8	19.8	0.5	0.3	18.5	19.6	0.7	0.4	18.4	19.7	0.5	0.3	21.3	21.9	0.4	0.3	19.4	21.4	1.4	0.3
7	3, 3, 4, 8	84.41	197696.9	28.4	29.9	0.9	0.3	28.4	30.0	0.9	0.4	28.6	30.0	0.7	0.3	28.4	29.5	0.8	0.4	28.6	30.7	1.2	0.4
8	5,5,40,239	n/a	n/a	2359.4	2675.5	150.8	51.5	23.9	343.5	222.4	55.7	0.0	21.6	29.4	50.9	0.0	33.6	33.3	51.3	0.0	0.0	0.0	11.7
9	5,5,40,247	n/a	n/a	2779.6	3315.4	189.1	55.2	6.6	340.2	271.4	56.1	0.0	32.7	31.6	53.9	1.9	289.6	144.8	54.6	0.0	1.2	3.8	24.1
10	5,5,40,250	n/a	n/a	1724.2	2113.6	219.9	58.0	4.7	518.4	410.5	57.3	0.0	4.6	6.8	55.1	0.0	8.3	10.8	55.9	0.0	0.0	0.0	9.7
11	5,5,40,256	n/a	n/a	3327.2	3831.0	210.6	59.9	104.5	757.9	399.2	59.1	2.6	64.9	52.8	57.6	23.9	307.3	156.0	58.9	0.0	0.0	0.0	14.8
12	5,5,40,264	n/a	n/a	2615.2	3010.5	217.4	61.2	62.1	464.9	292.9	64.6	0.0	27.1	29.2	60.3	0.0	129.3	163.8	60.8	0.0	0.0	0.0	13.1
13	8,8,60,328	n/a	n/a	1788.8	2183.7	182.5	89.8	12.9	426.6	370.3	91.6	22.1	606.8	405.3	92.3	0.0	178.1	172.2	61.3	0.0	0.0	0.0	22.3
14	8,8,60,370	n/a	n/a	5263.1	6144.6	443.3	108.9	1781.7	3441.9	924.6	104.9	1978.3	3072.9	706.9	110.2	1019.2	2641.6	858.3	109.4	0.0	1.5	6.4	42.2
15	10,10,80,518	n/a	n/a	12247.5	14265.1	919.1	197.3	7828.5	11064.3	1635.4	202.8	9015.9	10928.3	1004.5	198.8	6465.9	9917.7	1356.9	198.6	0.0	71.3	167.2	139.6

Notes: (1) n/a was due to LINGO running out of working memory to generate the model. (2) All customer orders in instances 1, 4, 8, 9, 11, 12, and 14 could use any transferring equipment. (3) Some customer orders in instances 2, 3, 5, 6, 7, 10, 13, and 15 could not use some of transferring equipment.

**Table 7:** The *t*-values of average total tardiness from the one-sided *t*-test.

Ins	Problem size	*t*-value of avg total	*t*-value of avg total
	(*m*, *q*, *n*, *o*)	*μ*_DE_ − *μ*_SSDE_	*μ*_RAM-EPSDE_ − *μ*_SSDE_
8	5-5-40-239	4.02	5.53
9	5-5-40-247	5.42	10.91
10	5-5-40-250	3.71	4.21
11	5-5-40-256	6.73	10.79
12	5-5-40-264	5.08	4.32
13	8-8-60-328	8.20	5.66
14	8-8-60-370	23.80	16.85
15	10-10-80-518	58.40	39.45

## Data Availability

Access to data is restricted due to third party rights.
